# Adipocyte abundances of CES1, CRYAB, ENO1 and GANAB are modified *in-vitro* by glucose restriction and are associated with cellular remodelling during weight regain

**DOI:** 10.1080/21623945.2019.1608757

**Published:** 2019-04-30

**Authors:** Qi Qiao, Freek G. Bouwman, Marleen A. van Baak, Nadia J. T. Roumans, Roel G. Vink, Susan L. M. Coort, Johan W. Renes, Edwin C. M. Mariman

**Affiliations:** a Department of Human Biology, NUTRIM School of Nutrition and Translational Research in Metabolism, Maastricht University Medical Centre, Maastricht, The Netherlands; b Institute for Technology-Inspired Regenerative Medicine, MERLN, Maastricht University Medical Centre, Maastricht, The Netherlands; c Department of Bioinformatics, NUTRIM School of Nutrition and Translational Research in Metabolism, Maastricht University Medical Centre, Maastricht, The Netherlands

**Keywords:** *In-vitro* fat-regain, SGBS adipocytes, proteomics, weight regain, focal adhesion

## Abstract

Long-term weight loss maintenance is a problem of overweight and obesity. Changes of gene expression during weight loss (WL) by calorie restriction (CR) are linked to the risk of weight regain (WR). However, detailed information on genes/proteins involved in the mechanism is still lacking. Therefore, we developed an *in-vitro* model system for glucose restriction (GR) and refeeding (RF) to uncover proteome differences between GR with RF vs normal feeding, of which we explored the relation with WR after WL. Human Simpson-Golabi-Behmel Syndrome cells were subjected to changing levels of glucose to mimic the condition of CR and RF. Proteome profiling was performed by liquid chromatography tandem mass spectrometry. This *in-vitro* model revealed 44 proteins differentially expressed after GR and RF versus feeding including proteins of the focal adhesions. Four proteins showed a persistent up- or down-regulation: liver carboxylesterase (CES1), mitochondrial superoxide dismutase [Mn] (SOD2), alpha-crystallin B-chain (CRYAB), alpha-enolase (ENO1). *In-vivo* weight loss-induced RNA expression changes linked CES1, CRYAB and ENO1 to WR. Moreover, of these 44 proteins, CES1 and glucosidase II alpha subunit (GANAB) during follow up correlated with WR. Correlation clustering of *in-vivo* protein expression data indicated an interaction of these proteins with structural components of the focal adhesions and cytoplasmic filaments in the adipocytes.

## Introduction

Overweight and obesity are major risk factors for various health complications like type II diabetes, cardiovascular disorders, sleep apnoea and certain types of cancer [1–4]. The prevalence of overweight and obesity is increasing worldwide and to date not a single country has successfully reversed its epidemic [5–7]. Remedies to obesity are losing weight by dietary intervention, increased physical activity, pharmacological treatment or surgical treatment [1,8–10]. However, up to 80% of individuals who lose weight on a low energy diet usually regain weight and often return to their original weight or even beyond it, within one or two years [2,11–17]. The common phenomenon of weight regain not only makes reduction of body weight less efficient, but also appears to induce an increase in the risk of metabolic complications [18]. As such, weight cycling is a core problem of overweight and obesity [5,14,19]. Therefore, it is important to gain more knowledge on the conditions and mechanisms of weight regain after weight loss in order to preserve reduced weight and the accompanying health improvements.

Various factors such as psychosocial and lifestyle influences are involved in regaining weight [12,20], but recent studies have led to a growing awareness on the involvement of physiological and molecular parameters related to the adipose tissue and the adipocytes [21–23]. Changes of gene expression during weight loss by calorie restriction (CR) and during the first weeks after return to energy balance were linked to the risk for weight regain. This involved in particular genes for components of the extracellular matrix or genes responsive to cell stress [24]. In order to find additional mechanistic leads, we decided to study the proteome of adipocytes *in-vitro* and compare normal feeding with refeeding (RF) after GR. Human Simpson-Golabi-Behmel Syndrome (SGBS) cells have proven to be an optimal and unique model for studying human adipocyte biology because of their morphological, biochemical and functional similarity to primary human adipocytes [25–27]. Here we have subjected SGBS adipocytes to glucose restriction (GR) and RF, and studied the changes of the cellular proteome in conjunction with changes in fat droplet size. Findings of proteins differentially regulated in this *in-vitro* study were compared with data from a previous *in-vivo* study, referred to as the Yoyo-study, in which weight regain after weight loss was investigated [19,24].

## Results

### The morphology changes of adipocyte fat droplets during glucose restriction and refeeding

**Figure 1.**
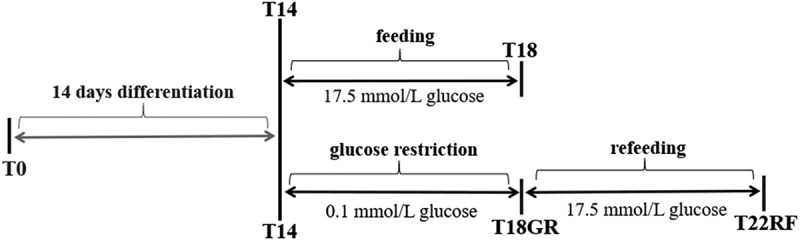
Schematic overview of the study design. SGBS pre-adipocytes at time point T0 were differentiated for 14 d. Then, mature adipocytes were used to start a control group for normal feeding (4 d) and a test group for glucose restriction (4 d) and refeeding (4 d). GR: glucose restriction, RF: refeeding.

**Figure 2.**
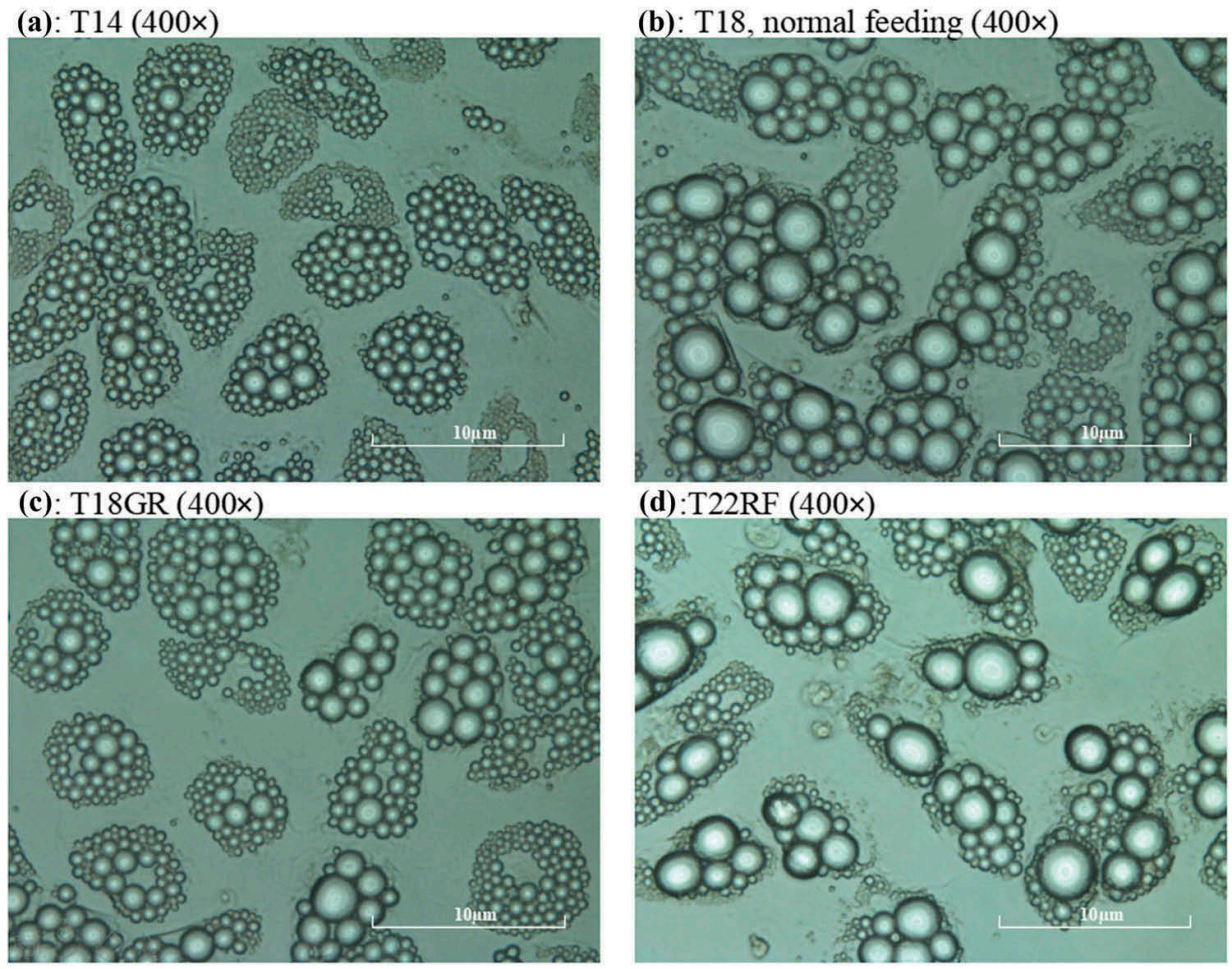
Recording of mature SGBS adipocytes during the experiment. a: mature adipocytes at time point T14, b: adipocytes at T18, c: adipocytes at T18GR, d: adipocytes at T22RF. a, b, c, d are all shown at 400 × magnification by the microscope camera system. GR: glucose restriction, RF: refeeding.

**Figure 3.**
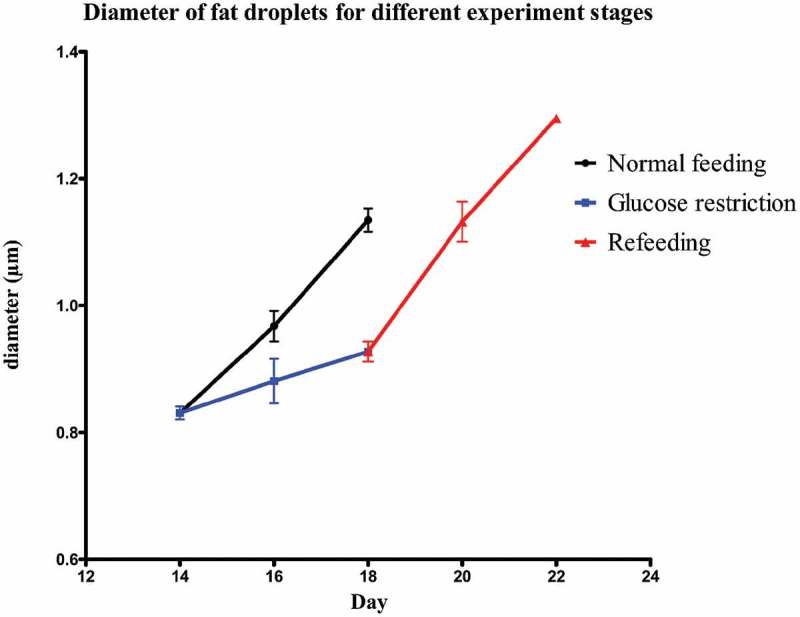
Diameter of fat droplets for the different experimental stages from T14 onwards.

Mature SGBS adipocytes were treated according to the experiment overview of Figure 1. On day 14 (T14) 85–88% pre-adipocytes had differentiated into mature adipocytes, which were occupied by fat droplets. The average diameter of the five biggest droplets per adipocyte was 0.83 ± 0.01 µm. The control group was maintained for 4 d on 17.5 mmol/L glucose, which led to an increase in fat droplet size. On day 16 the diameter had significantly (*P* = 0.01) increased to 0.97 ± 0.01 µm and it continued to increase significantly (*P* = 0.01) to 1.13 ± 0.01 µm on day 18 (T18). The test group underwent GR for 4 d with 0.1 mmol/L glucose. Despite the glucose restriction, the diameter increased slightly up to 0.88 ± 0.02 µm on day 16 and 0.93 ± 0.01 µm on day 18 (T18GR). Both these changes (T14-T16GR and T16GR-T18GR) were not significant (*P* = 0.26 and *P* = 0.24, respectively), whereas the 4 d change (T14-T18GR) reached borderline significance (*P* = 0.05). After 4 d GR, the medium was adjusted to 17.5 mmol/L glucose and the cells were cultured for another 4 d. In the first two days, the diameter of fat droplets took a sharp rise, going up from 0.93 ± 0.01 µm at T18GR to 1.13 ± 0.02 µm on day 20 (*P* = 0.007). It continued to increase to 1.30 ± 0.001 µm on day 22 (T22RF) (*P* = 0.036). Figure 2 shows images of the cells at different time points. The diameter values have been graphically depicted in Figure 3 (see also Supplemental Table 1). This analysis shows that the increase of fat droplet size follows similar kinetics during the 4 d feeding (T14-T18) and 4 d RF period (T18GR-T22RF) suggesting a rapid return to the normal metabolism after GR. The increase in diameter was 0.30 µm for T14-T18 and 0.37 µm for T18GR-T22RF, respectively. Although not significant (*P* = 0.07), it may suggest that GR stimulates fat droplet size resulting in a larger diameter of the fat droplets after RF. In line with a higher fat droplet diameter after GR and RF, the OD values after ORO staining were 1.55 ± 0.03 at T18 after 4 d normal feeding and 1.69 ± 0.03 after 4 d GR and 4 d RF (*P* = 0.13) (Supplemental Table 1).

### Proteomics analysis of glucose restriction (T14 vs T18GR)

**Figure 4.**
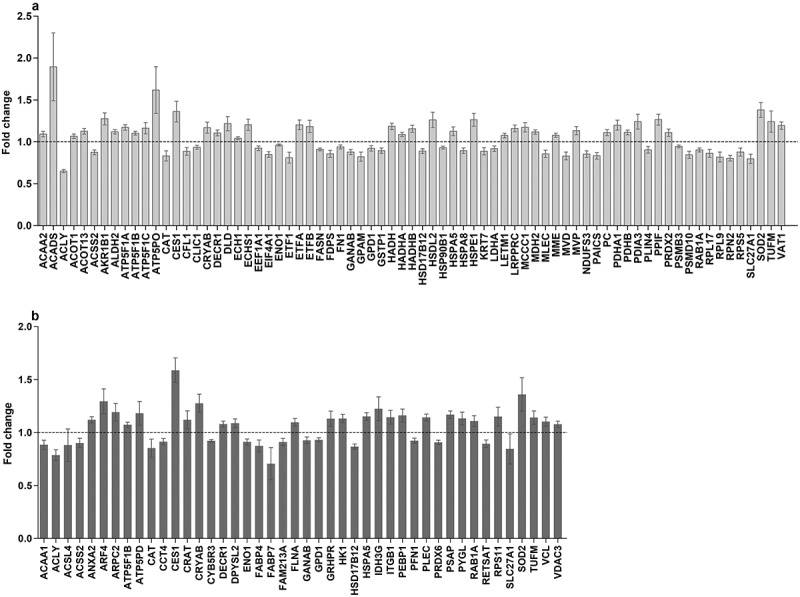
*In-vitro* proteomic changes in adipocytes at different time period. (a**)**: Proteins significantly changed during GR. (b**)**: Proteins significantly changed during RF after GR versus feeding. Dependent T-test was used for normal distributed variables, otherwise Wilcoxon test was used. *P* < 0.05 was considered significantly different. GR: glucose restriction, RF: refeeding.

Because during the 4 d GR, the diameter of the fat droplets still seemed somewhat to increase, we were curious to see if and how the cells had changed their metabolism. Proteomics analysis revealed that from a total of 393 proteins, 73 proteins had changed significantly after 4 d GR, 39 increased and 34 decreased (Figure 4(a), Supplemental Table 2). Using the log_2_ (FC) and *P* values of the 393 proteins for pathway analysis, 28 pathways were picked up according to the PathVisio over-representation analysis (Supplemental Table 3; Z score >1.96, *P* < 0.05) [28], but only 12 pathways had more than 1 measured protein. More detailed analysis of these data showed that beta-oxidation is up-regulated (Supplemental Figure 1) and de novo fatty acid synthesis is down-regulated (Supplemental Figure 2) during GR. Despite the slight increase of fat droplet size, after 4 d the metabolism of the adipocytes seems to have responded as expected to the restriction of glucose and adjusted the fatty acid handling. Further, we observed a significant up-regulation of various subunits of the F1-complex and Stalk of the Complex V ATP synthase (Supplemental Figure 3). An increase in the synthesis of ATP through the electric transport chain may compensate for the loss of ATP production during glycolysis.

### Proteomic comparison of feeding and refeeding: T18 vs T22RF

Since the 4 d RF shows similar kinetics as the 4 d feeding for the increase of the measured fat droplet size, it can be expected that the cellular metabolism after GR has re-adjusted to the normal feeding condition. As such, differences in protein abundance between T18 and T22RF will reflect the sustained effects of GR after return to normal feeding. Proteome analysis of the RF phase showed that most proteins that were significantly up- or down-regulated during GR, reversed their expression during RF in line with a return to the normal feeding state. For instance, fatty acid synthase (FASN), the driver of de novo fatty acid synthesis, was significantly down-regulated during GR and significantly up-regulated during RF. Comparing the proteome of T18 and T22RF revealed 44 significantly different proteins (Figure 4(b), Supplemental Table 4). Over-representation analysis revealed six significant pathways with a coverage between 4% and 43% with at least two differential proteins. One of these pathways is ‘Integrin-mediated Cell Adhesion’. In fact, 5 of the 44 differential proteins are closely associated with focal adhesions: integrin beta-1 (ITGB1), vinculin (VCL), filamin-A (FLNA), plectin (PLEC) and profilin (PFN1). The first four are significantly up-regulated, whereas profilin is down-regulated. It suggests a profound dynamics of the focal adhesions induced by GR preceding normal feeding.

Of the 44 differential proteins, 9 proteins had a significant change in abundance during GR (T14-T18GR) as well as during RF (T18GR-T22RF). Five of them showed the opposite changes during GR and RF suggesting that they were in the process of readjusting to the normal feeding state. Of the other four proteins, three showed a constitutive up-regulation: liver carboxylesterase (CES1), mitochondrial superoxide dismutase [Mn] (SOD2), alpha-crystallin B-chain (CRYAB), and one showed down-regulation: alpha-enolase (ENO1). As these four proteins appeared to have their expression reset by GR, we decided to investigate their relation with weight regain.

### Assessing the link between constitutively regulated proteins with weight regain in-vivo

In order to assess the link between these four proteins and weight regain, we examined gene and protein expression data of the Yoyo study [19] (see Methods section). As can be seen in Table 1 the change in RNA expression during the intervention (T3-T1) is significantly correlated with the percentage weight regain for CES1, CRYAB and ENO1. At the protein level, only CES1 was significantly correlated with percentage weight regain (r = 0.397, *P* = 0.012), not for the change during the intervention (T3-T1) but during the follow-up (T4-T3). In addition, 29 of the 44 proteins for which there was information in the Yoyo study, besides CES1 also Glucosidase II alpha subunit (GANAB) was correlated with WR during follow-up (r = 0.47, *P* = 0.02).

**Table 1.** Correlations of RNA and protein changes with weight regain percentage.

	RNA (T3-T1)			Protein (T3-T1)			Protein (T4-T3)	
Names of value	*r* value	*P* value		*r* value	*P* value		*r* value	*P* value
CES1	0.56	< 0.001		0.24	0.13		0.397	0.01
CRYAB	0.54	< 0.001		0.07	0.67		−0.10	0.53
SOD2	0.15	0.31		0.04	0.82		−0.17	0.31
ENO1	0.42	0.002		0.01	0.53		0.07	0.69

Weight regain percentage: ((weight after follow up T4 – weight after WS T3）÷ weight after WS T3) × 100%. Correlation coefficients (*r* value) was calculated by Spearman Rho’s; *P* value < 0.05 was considered as significantly correlated.

### Correlation clustering of CES1, CRYAB, ENO1 and GANAB with adhesome proteins

**Figure 5.**
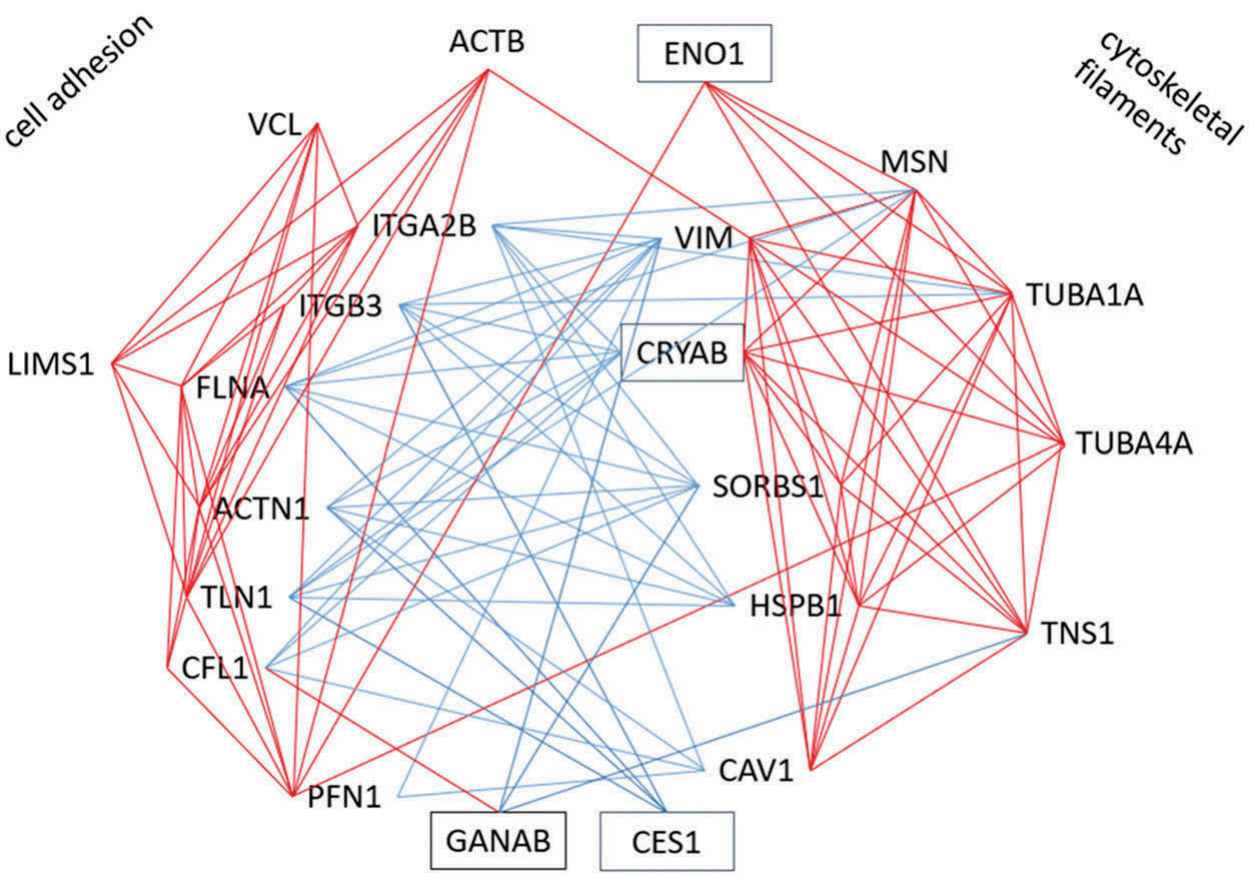
Cluster of significant correlations of CES1, CRYAB, ENO1 and GANAB with adhesome proteins in Yoyo study. Red line indicates significant positive correlation; blue line indicates significant negative correlation.

A function for CES1, CRYAB, ENO1 and GANAB in weight regain has not been proposed, but a role for focal adhesions has been suggested [24]. Therefore, we tried to find out whether there was a link between these proteins and the focal adhesion complex. For that purpose, we constructed a co-correlation plot (T4-T3) between these four proteins and 37 proteins of the focal adhesome [29] that were identified in the Yoyo study (for gene names see Supplemental Table 5). Hierarchical clustering revealed several interacting groups. Focusing on significant correlations (Figure 5), this showed that CES1 and CRYAB correlate negatively with integrin beta-3 (ITGB3), FLNA, alpha-actinin (ACTN1) and talin (TLN1). Other proteins that negatively correlated with these four proteins are sorbin and SH3 domain containing 1 (SORBS1), heat shock protein 27 (HSPB1/HSP27) and vimentin (VIM). Further, GANAB correlated positively with cofilin (CFL1) and negatively with tensin-1 (TNS1), SORB1 and VIM. Notably, ITGB3, FLNA, ACTN1 and TLN1 correlate positively with each other and with vinculin (VCL), PFN1, CFL1, beta-actin (ACTB), integrin alpha-2B (ITGA2B) and LIM zinc finger containing 1 (LIMS1). CRYAB correlates with 20 of the 37 investigated adhesome proteins. CRYAB and ENO1 correlate positively with tubulin alpha 1A (TUBA1A), tubulin alpha 4A (TUBA4A), moesin (MSN) and TNS1. Other proteins that correlate positively with the latter four proteins are HSPB1/HSP27, VIM, SORBS1 and caveolin (CAV1). These correlations were used to construct an interaction plot (see Figure 5).

## Discussion

In the present study, we established an *in-vitro* model of GR and RF of SGBS adipocytes, investigated morphologic characteristics of adipocytes and fat droplets and analysed the proteome changes during GR and RF. We observed that the RF phase closely mimicked the feeding phase with respect to the growth rate of the largest fat droplets, allowing us to assess the influence of GR before RF. We intended to uncover proteins of which the abundance in adipocytes was significantly different between 4 d feeding and 4 d RF. These proteins can be regarded as candidates for metabolic consequences of CR *in-vivo* including the risk for weight regain and cardiometabolic complications. Forty-four differential proteins were detected and four of them significantly changed expression in the same direction during GR and RF. *In-vivo*, significant positive correlations were detected with weight regain percentage for CES1, CRYAB and ENO1 at the RNA level during the diet intervention (T3 to T1), and for CES1 and GANAB at the protein level during follow up (T4 to T3). In fact, ENO1 and CRYAB have been reported earlier as part of stress gene interaction networks in relation to weight regain [19,30]. This supports our *in-vitro* strategy to identify relevant factors for metabolic consequences of CR.

Carboxylesterase 1 is a serine esterase which functions in the detoxification of xenobiotics [31]. Studies have indicated that environmental pollution could be a risk factor for obesity [32,33], although the evidence is not conclusive [34]. It may therefore be hypothesized that certain pollutants or food contaminants may contribute to the risk for weight regain after weight loss. People living in a more polluted area may retain higher levels of CES1 in the adipose tissue than people in a cleaner environment. According to our present observations, this might result in a higher risk of weight regain.

Besides xenobiotic detoxification, CES1 seems to have various functions in the normal physiology of mammals [35]. In the mouse, its homologue Ces3 has been demonstrated to have triacylglycerol hydrolase activity [36]. Such an activity would be in line with the up-regulation that we observed during the glucose restriction. However, Jernas et al. [37] have shown that in humans CES1 gene expression reduces during CR and is linked to body fat and adipocyte fat content instead of lipolytic activity. Similarly, in the Yoyo study during the dietary intervention (T3-T1) both the RNA (FC = −1.56, *P* < 10^–4^) and the protein (FC = −1.22, *P* < 0.001) decreased [19]. It suggests that individuals, in whom the CES1 RNA and/or protein is less reduced during the dietary intervention, have a higher risk of weight regain. This fat-preserving activity of CES1 is in line with the fact that mRNA levels in adipose tissue positively correlate with BMI and triglycerides besides cardio- and glucometabolic parameters [38].

CRYAB is known as a component of the eye lens and as a chaperone protein involved in the regulation of oxidative and endoplasmic reticulum stress [39], but it may still have other functions in adipocytes. Stefan et al. [40] validated CRYAB as a novel adipokine and illustrated that its expression is strongly induced during adipogenesis, reaching a 10 times higher level in mature adipocytes than in pre-adipocytes. In adipocytes from obese patients, its abundance increases with age suggesting that it may be involved in obesity-related complications.

ENO1 is a well-known enzyme of the glycolysis. Hexokinase 1 and glycogen phosphorylase are higher at T22RF, which suggests an up-regulation of the glucose conversion after the GR. This is in keeping with a higher abundance of annexin 2, which serves in the GLUT4 translocation to the membrane [41]. Boosting the metabolism by glucose without supplying fatty acids may explain the significantly lower level of fatty acid binding proteins 4 and 7, with consequences for the cellular fatty acid metabolism. De novo fatty acid synthesis is down-regulated during GR, but recovers during the RF phase. The persistent decrease of ENO1 during GR and RF seems in contrast to an increased glucose conversion. It suggests that ENO1 may serve yet another function in adipocytes.

GANAB is a key glycoprotein quality control protein in endoplasmic reticulum, where it removes glucose residues from immature glycoproteins [42]. Mutations in the gene can cause polycystic kidney disease, but its roles for weight regain has not been raised yet.

Based on the annotated functions of CES1, CRYAB, ENO1 and GANAB, it is not clear what processes they favour during GR and RF *in-vitro* or during weight regain after weight loss *in-vivo*. Arguments have been presented that adipocyte stress which accumulates during fat loss, is a driving factor for fat regain during follow-up after return to energy balance [23]. This mechanical stress is concentrated at the focal adhesions linking the stress-fibres of the cytoplasm to the extracellular matrix [43–46]. In the present *in-vitro* study, we also observed a higher level of components of the focal adhesion when RF was compared to normal feeding. For instance, higher levels were seen of VCL, FLNA, ITGB1, PFN1, plectin and actin-related protein 2/3 complex subunit 2. Whereas VCL, FLNA and ITGB1 are core proteins of the focal adhesion complex, the other proteins bind to it. In 3T3L1 adipocytes and to some extent in SGBS cells, plectin has been shown to be part of the nesprin-3/plectin/vimentin complex that forms a network around lipid droplets [47].

We wondered if we could find a link between CES1, CRYAB, ENO1 and GANAB on one hand, and focal adhesions on the other. Using changes of protein abundance (T3-T4) of the Yoyo study, a network of significant correlations was constructed from these four proteins and 19 proteins belonging to intrinsic or peripheral components of the adhesome (Figure 5). This led to two positive clusters, of which one seems in function closer to the focal adhesion whereas the other has more components of structural filaments. These two clusters are mainly linked by negative correlations. Interestingly, CES1, CRYAB, ENO1 and GANAB interact with these clusters: CES1 correlates negatively with the adhesion cluster, ENO1 positive with the filament cluster, CRYAB negatively with the adhesion and positive with the filament cluster, GANAB negatively with cytoskeletal filaments. This suggests that CES1, CRYAB, ENO1 and GANAB function in the intracellular reorganization of focal adhesions and cytoskeletal filaments. It can be hypothesized that in response to CR these proteins modify the adipocyte structural organization with an impact on the risk for weight regain.

It should be noticed that CR in the present *in-vitro* study was merely the result of GR. In addition, we kept the glucose concentration during the feeding (T14-T18) and RF (T18GR-T22RF) states at 17.5 mmol/L, which is the optimal level for SGBS differentiation and growth, but which poorly reflects the human physiological situation. Despite the rather strict conditions of GR, the diameter of the five biggest fat droplets per adipocyte slightly continued to grow. This may be due to nutrient resources available in the cells, but also may reflect droplet-fusion [48]. Nevertheless, pathways of fatty acid and glucose handling responded as expected to CR and RF.

## Conclusions

In this paper, we have reported on the *in-vitro* identification of proteins of human SGBS adipocytes with a GR-modified expression level. CES1, CRYAB and ENO1 are persistently up- or down-regulated during the GR-RF intervention. Correlation clustering revealed that, together with GANAB, these proteins interact with core and peripheral proteins of the focal adhesion complex and structural filaments. It suggests a role of these proteins in structural remodelling of the adipocytes. *In-vivo* data analysis links these proteins to weight regain after weight loss. Since these findings are only suggestive, further research is needed to investigate their function in adipocytes in more detail and also their relevance for weight regulation.

## Methods

### Study design

SGBS pre-adipocytes at time point T0 went through a 14 d differentiation process (Figure 1). Mature adipocytes on day 14 (T14) were randomly divided into two research lines: control group and test group. For the control group, SGBS adipocytes were cultured under normal feeding conditions for 4 d till day 18 (T18). For the test group, SGBS adipocytes first went through a 4 d condition of low-glucose (glucose restriction till day 18, T18GR), subsequently the medium was switched to normal feeding conditions for another 4 d (RF till day 22, T22RF). Cell growth was closely monitored by microscopy, and fat droplets were recorded every second day from T14 onwards to give information on morphologic changes during GR and RF. Cellular proteins were isolated at time points T14, T18, T18GR and T22RF. Finally, proteome profiling was performed by liquid chromatography tandem mass spectrometry (LC-MS/MS) to uncover cellular processes involved in the different stages of the SGBS cells. The entire experiment was repeated three times and for each experiment cell culture was performed in triplicate.

### Cell culture and induction of differentiation

Human SGBS pre-adipocytes of passage 7 were cultured in Gibco™ Dulbecco’s Modified Eagle Medium: Nutrient Mixture F-12 (DMEM/F-12) Media (Life Technologies) supplemented with 66 mmol/L biotin, 34 mmol/L D-pantothenate (Sigma-Aldrich), 10% fetal calf serum (Bodinco BV) and 1% penicillin and streptomycin (Life Technologies) as described before [27]. At passage 9 cells were seeded in 6-well plates (Corning Life Sciences, Amsterdam, The Netherlands) with the amount of 3 × 10^4^ cells per well. Every two days, the medium was refreshed. Once the pre-adipocytes reached 90% confluence (T0), the medium was changed to serum-free DMEM/F12 differentiation medium containing 2 mg/mL human transferrin, 200 µmol/L human insulin, 5 mmol/L cortisol, 20 µmol/L triiodothyronine, 1 mmol/L 3-isobutyl-1-methylxanthine and 5 mmol/L rosigilitazone (Sigma-Aldrich). After 4 d the medium was changed to serum-free DMEM/F12 medium containing 2 mg/mL human transferrin, 200 µmol/L human insulin, 5 mmol/L Cortisol, 20 µmol/L triiodothyronine. Every second day, the differentiation medium was refreshed. After 14 d, 85–88% of pre-adipocytes were differentiated into mature adipocytes (T14). To determine cell numbers, pre-adipocytes were trypsinized and counted with a haemocytometer (Countess), adipocytes were counted using a raster ocular.

### Glucose restriction and refeeding

For GR experiments, mature adipocytes (T14) were cultured in DMEM/F12 (1:1) without glucose and phenol red (Cell Culture Technologies), supplemented with 20 nmol/L human insulin and 0.1 mmol/L glucose for 96 h (T18GR). As control, mature adipocytes (T14) originating from the same pre-adipocytes were cultured 96 h in normal feeding medium: the same DMEM/F12 medium supplemented with 20 nmol/L human insulin and 17.5 mmol/L D-glucose (T18) [49].

For RF experiments, after 96 h under GR, the cells were cultured in DMEM/F12 medium supplemented with 20 nmol/L human insulin and 17.5 mmol/L D-glucose for another 96 h (T22RF). From T14 onwards, the medium was gently refreshed every second day.

### Monitoring fat droplet size

The average diameter of all measurable fat droplets is an underrepresentation of the average diameter of all fat droplets. Therefore, we decided to determine the mean diameter of the five biggest fat droplets as a parameter related to the turnover of stored fat during feeding, GR and RF [50]. In detail, cell growth was closely recorded from T0 onwards using a Nikon Eclipse TS100 microscope equipped with a Digital Sight microscope camera control unit (DS-L3), (Nikon). After day 14, every second day approximately 300 adipocytes were randomly chosen on the image. Meanwhile, in each figure recording at 400 × magnification, the five biggest fat droplets per cell were selected by eye and their diameters were measured. If selection by eye was not possible, the diameters of the eight biggest droplets were measured and the five biggest ones were selected based on the measured values. Finally, we calculated the mean diameter of the five biggest fat droplets.

### Oil Red O (ORO) staining

Cells were washed twice with PBS, then incubated with 3.7% formaldehyde for 1 h. During incubation, the number of adipocytes was determined using a raster ocular. After rinsing the cells with Milli-Q and 70% ethanol, the liquid was aspirated completely. 0.5 gram Oil Red O (Sigma-Aldrich) was dissolved in 50 mL isopropanol (Sigma-Aldrich), 30 mL of this solution was mixed with 20 mL Milli-Q water and filtered through a 0.2 µmol/L device. Aspirated cells were stained with this ORO working solution for 30 min. After staining, cells were washed 8 times 30 s with 70% ethanol, then washed twice with Milli-Q for 5 min and finally cells were dissolved in 2 mL DMSO (Sigma-Aldrich). The OD value was determined at 540 nm. The ORO value was corrected for the number of cells as follows: OD _corrected value_ = (OD _measured value_/amount of cells) × 10^5^.

### Protein isolation

For protein isolation, cells were collected at each of the above time points for both the control and test group. In detail, wells with cultured cells were washed twice with PBS buffer and lysed with SDT buffer (2% sodium dodecyl sulfate/50 mmol/L dithiothreitol/100 mmol/L Tris-HCl pH = 7.6), 300 µL per well. Cells were scraped off with a cell scraper (Corning) and the lysate was collected in tubes, then heated at 95°C for 5 min. After heating, samples were sonicated in three 20-s cycles and centrifuged at 16000 × g for 5 min at 20°C, and then the supernatant was carefully transferred to another tube. All samples were stored at −80°C for protein digestion and LC-MS/MS. The entire experiment was performed three times and for each experiment 3 wells of cells were available for each protein isolation at all the time points.

### Protein sample preparation and digestion

Amicon Ultra 0.5 mL centrifugal filter devices (Sigma-Aldrich) were soaked overnight with 5% Tween 20. Filter devices were washed by immersion in Milli-Q for 10 min with 600 rpm shaking, then 500 µL Milli-Q was added and filter devices were centrifuged at 14000 × g at 20°C for 25 min. Subsequently, the filter unit was inserted in a filtrate collection vial. Protein sample was added into the filter unit, centrifuged at 14000 × g at 20°C for 30 min. After the solution in the collection vial was discarded, the filter was inverted and centrifuged at 2000 rpm at 20°C for 2 min.

For alkylation, 50 µL of the filter-concentrated sample was mixed with 50 mmol/L iodoacetamide in a volume of 500 µL, and incubated for 30 min in the dark. The entire volume was transferred into the filter device and centrifuged at 14000 × g at 20°C for 30 min. Next, several steps were undertaken to remove sodium dodecyl sulfate and dithiothreitol. 500 µL of 8 mol/L urea supplemented with 4% sodium deoxycholate was added into the filter device and centrifuged at 14000 × g at 20°C for 45 min. This step was repeated twice with 500 µL of 8 mol/L urea and then twice with 50 mmol/L ammonium bicarbonate. Next, the concentrated protein sample was collected by inverting the filter unit and centrifugation at 1000 × g for 5 min. Protein concentration was determined using the microplate BCA protein assay according to the manufacturer’s protocol (Pierce, Thermo Fisher Scientific; 23252). For protein digestion, 42 µg protein of time points T14, T18, T18GR, T22RF was supplemented with 1 µg trypsin/Lys-C Mix (Thermo Fisher Scientific; V5073) and incubated for 9–14 h at 37°C.

### Sample desalting

After overnight digestion, the peptide samples were cleaned from residual sodium deoxycholate and SDS by precipitation with an equivalent volume of 4 mol/L potassium chloride, acidified to pH 1 to 2 with 2% formic acid. In detail, the samples were vortexed for 30 s, incubated for 5 min at room temperature to form precipitates, and subsequently centrifuged at 15700 × g for 15 min to pellet the precipitate. The supernatant was carefully removed and transferred to new tubes and acidified with 100% formic acid (FA) to pH 1 to 2 with pH paper. Then the peptide samples were desalted with a StageTip C18 column [51]. Briefly, the column made by stacking three layers of a 3 M Empore C18 anion exchange disk (Thermo Fisher Scientific) into a 10 µL micropipette tip, was pre-rinsed with 50 µL 70% acetonitrile (ACN) and equilibrated with 50 µL FA by air pressure. Then the sample was loaded on the column and eluted with 30 µL 70% ACN/5% FA, and the desalted sample was collected in a clean LoBind tube (Eppendorf, Sigma-Aldrich). Peptides were dried under vacuum (Concentrator plus, Eppendorf) and labelled with TMT 10plex Mass Tagging Kits (Thermo Fisher Scientific; 90111) according to the manufacturer’s protocol. In short, 42 µg peptides were diluted into 84 µL of 50 mmol/L triethyl ammonium bicarbonate. The labelling reaction was incubated for 1 h at room temperature and quenched 15 min by adding 8 µL of 5% hydroxylamine. Equal amounts of combined samples were transferred into a new micro-centrifuge tube for LC-MS/MS.

### Protein identification using LC-MS/MS

A nanoflow HPLC instrument (Ultimate 3000, Dionex) was coupled on-line to a Q Exactive mass-spectrometer (Thermo Scientific) with a nano-electrospray Flex ion source (Proxeon). The final concentration of the TMT labelled digest/peptide mixture was 0.33 μg/µL and 5 µL of this mixture was loaded onto a C18-reversed phase column (Thermo Scientific, Acclaim PepMap C18 column, 75-μm inner diameter x 15 cm, 2-μm particle size). The peptides were separated with a 120 min linear gradient of 4–68% buffer B (80% acetonitrile and 0.08% formic acid) at a flow rate of 300 nL/min.

MS data were acquired using a data-dependent top-10 method, dynamically choosing the most abundant precursor ions from the survey scan (280–1400 m/z) in positive mode. Survey scans were acquired at a resolution of 70,000 and a maximum injection time of 120 ms. Dynamic exclusion duration was 30 s. Isolation of precursors was performed with a 1.8 m/z window and a maximum injection time of 200 ms. Resolution for HCD spectra was set to 30,000, and the Normalized Collision Energy was 32 eV. The under-fill ratio was defined as 1.0%. The instrument was run with peptide recognition mode enabled, but exclusion of singly charged ions and charge states of more than five.

### Database search, quantification, normalization and pathway analysis

The MS data were searched using Proteome Discoverer 2.2 Sequest HT search engine (Thermo Scientiﬁc), against the UniProt human database. The false discovery rate (FDR) was set to 0.01 for proteins and peptides, which had to have a minimum length of six amino acids. The precursor mass tolerance was set at 10 ppm and the fragment tolerance at 0.02 Da. One miss-cleavage was tolerated, oxidation of methionine was set as a dynamic modification and carbamidomethylation of cysteines, TMT reagent adducts (+229.162932 Da) on lysine and peptide amino termini were set as fixed modifications. The data of each run were normalized to the total peptide amount in each channel and to compare between the runs scaled to time point T18. Quantified changes of identified proteins were used to perform pathway analysis and visualization by the PathVisio software version 3.3.0 [28,52].

### Data collection from in-vivo study

To compare the proteomic changes *in-vitro* and *in-vivo* and further explore the possible relation with weight regain, 53 human participants’ proteome quantification and microarray analysis data were extracted from the Yoyo study (registration number: NCT01559415 [53],). The process of data collection was shown in a flowchart (see Supplemental Figure 4). In short, the Yoyo study was a weight loss/follow-up intervention study on 61 overweight/obese persons. Anthropometric measurements were done, and samples including adipose tissue biopsies were taken before weight loss (T1), after a (very) low calorie diet for 5 weeks or 3 months to lose approximately 8% body weight (T2), after 4 weeks of being maintained on a balanced diet (T3), and after a 9-month follow-up period (T4). Protein quantification and microarray analysis were carried out as previously described [54]. Changes in RNA level from T1 to T3 were obtained from microarray analysis data. RNA data at T4 were not available. Changes in protein level from T1 to T4 were collected from protein quantification data.

### Statistical analyses

Data were presented as mean ± SEM. Statistical analyses were conducted using SPSS version 22.0 for Windows 10 (SPSS Inc., Chicago, IL, USA). Fold change (FC) was calculated during continued feeding, GR and RF, respectively. All variables were checked for normal distribution by Shapiro-Wilk’s test, *P* > 0.05 was considered as the threshold for normal distribution. For variables with normal distribution, paired t-test was used to compare values within a group at different time points; independent t-test was used for the comparison between control group and test group. For variables with a skewed distribution, Wilcoxon test was used. Spearman Rho’s correlation coefficients were calculated for relationships between parameters at different time points. In statistical analyses, *P* < 0.05 was considered to be significant, unless otherwise stated.
