# Supplementary figures and images for: Adipocyte abundances of CES1, CRYAB, ENO1 and GANAB are modified in-vitro by glucose restriction and are associated with cellular remodelling during weight regain
Source: Adipocyte. 2019 Apr 30;8(1):190–200. doi: 10.1080/21623945.2019.1608757 (PMC6768247; doi:10.1080/21623945.2019.1608757)

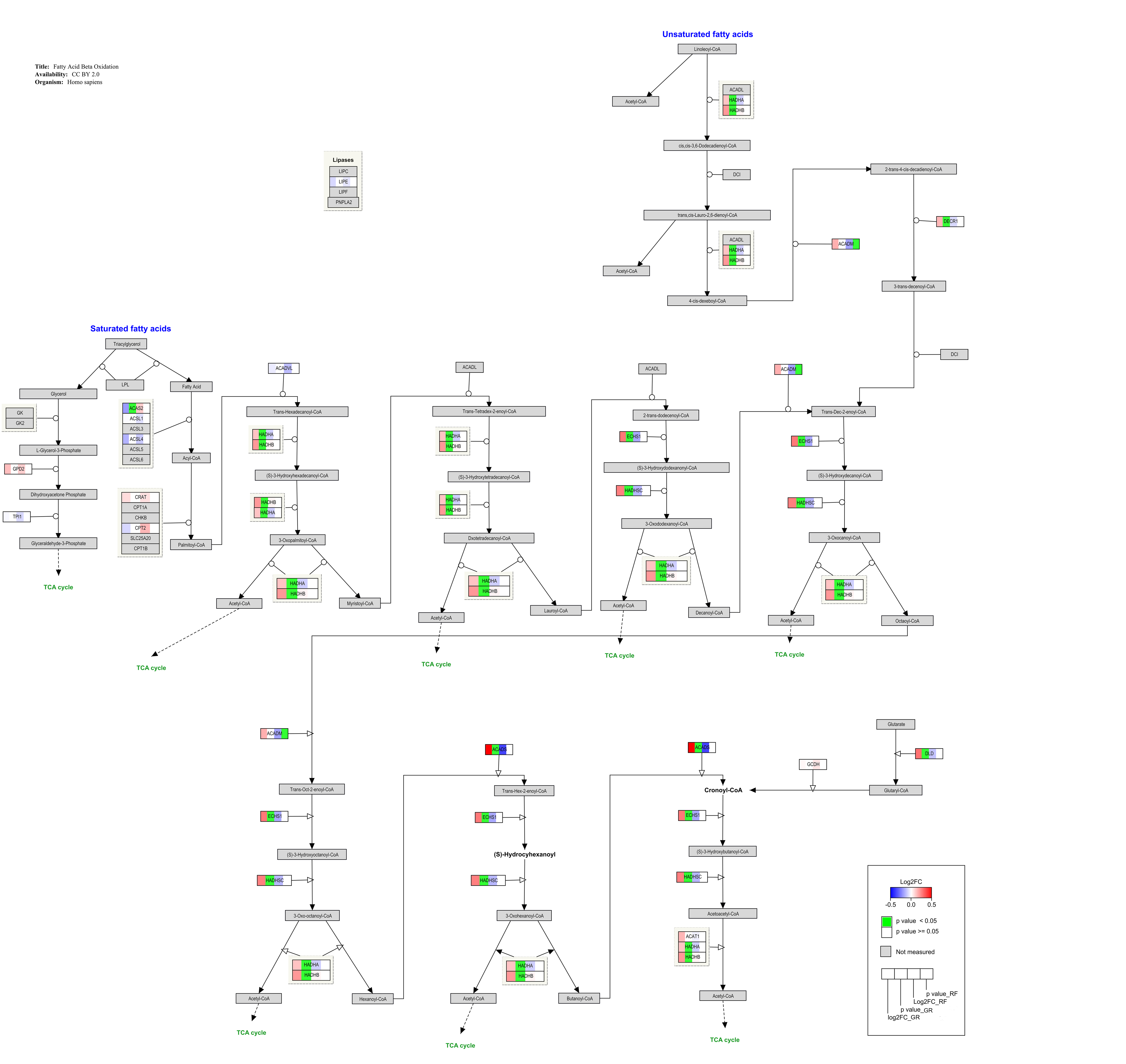

Supplement: Supplemental Material [file kadi-08-01-1608757-s001.zip › Supplemental Figure 1.tif]

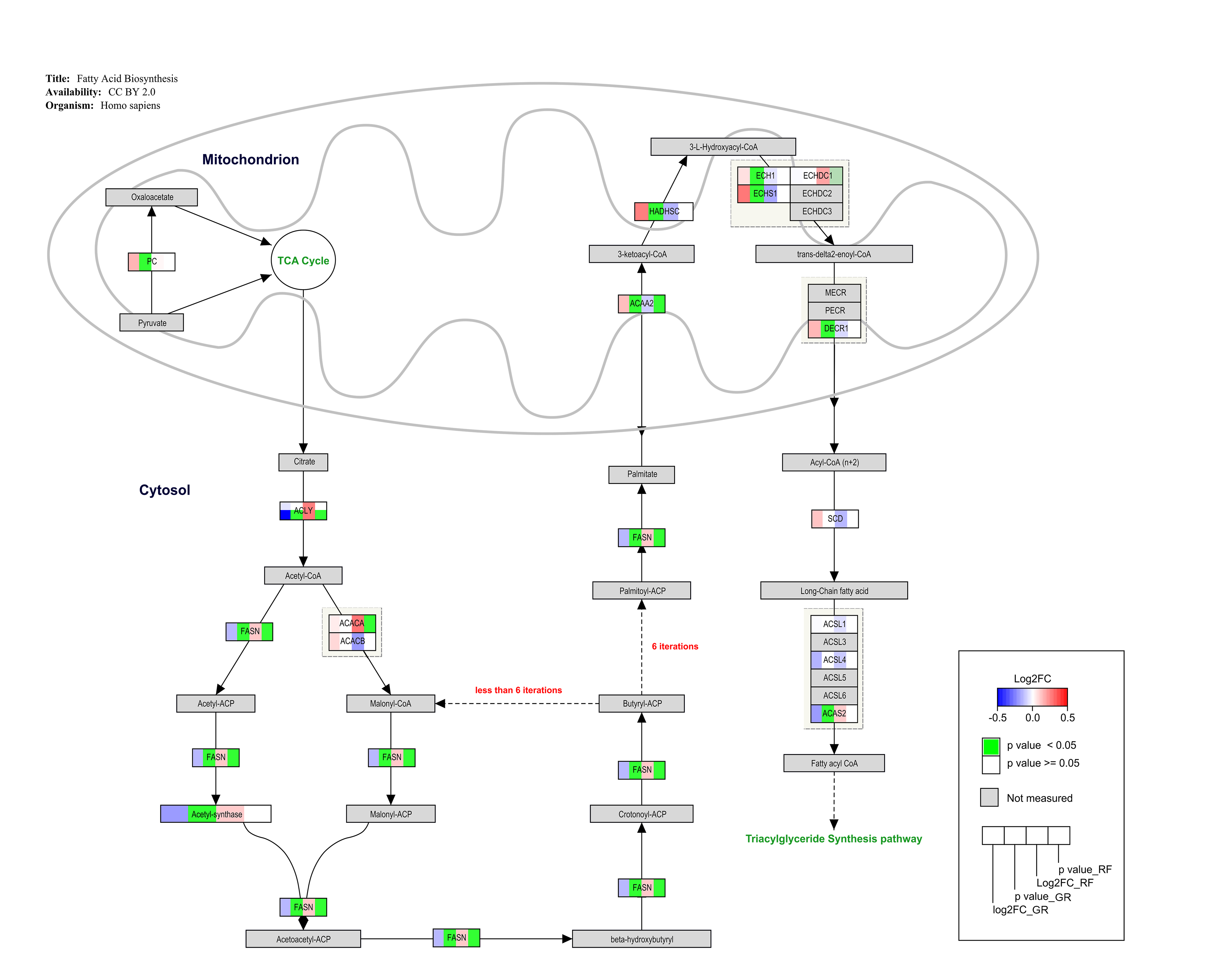

Supplement: Supplemental Material [file kadi-08-01-1608757-s001.zip › Supplemental Figure 2.tif]

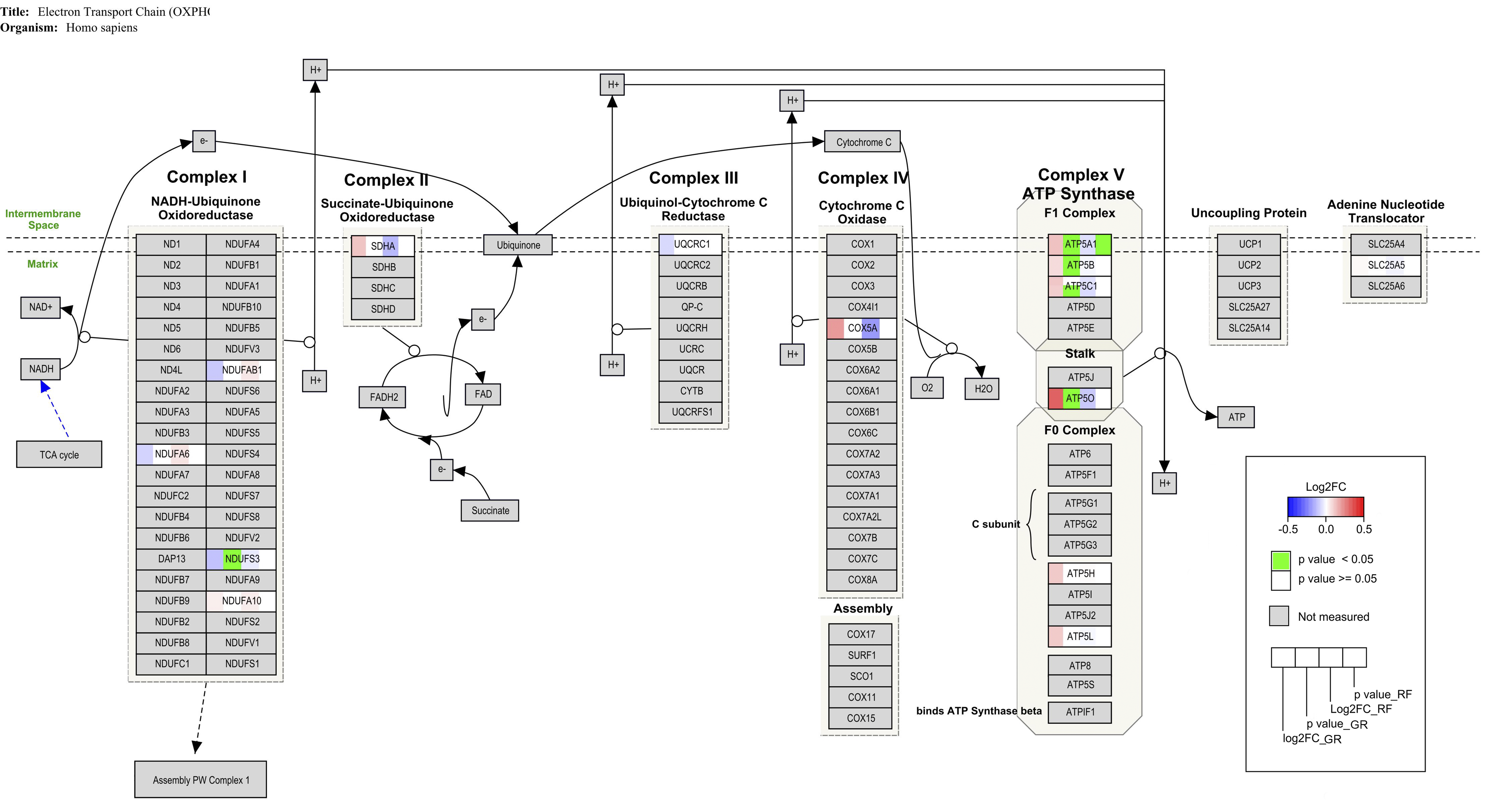

Supplement: Supplemental Material [file kadi-08-01-1608757-s001.zip › Supplemental Figure 3.tif]
