# Supplementary material for: Adipocyte abundances of CES1, CRYAB, ENO1 and GANAB are modified in-vitro by glucose restriction and are associated with cellular remodelling during weight regain
Source: Adipocyte. 2019 Apr 30;8(1):190–200. doi: 10.1080/21623945.2019.1608757 (PMC6768247; doi:10.1080/21623945.2019.1608757)
Supplement: Supplemental Material [file kadi-08-01-1608757-s001.zip › Supplemental Figure 4.doc]

**Allocation**

**Analysis**

**Follow-Up**

**Enrollment**

Assessed for eligibility (n= 156)

Excluded (n= 95)

♦  Not meeting inclusion criteria (n= 48)

♦  Declined to participate (n= 15)

♦  Other reasons (n= 32)

Analysed (n = 27)

♦ Excluded from analysis due to insufficient biopsy material (n = 2)

Lost to follow-up due to personal circumstances (n= 1)

Allocated to intervention (n= 30)

♦ Received allocated intervention (n= 30)

♦ Did not receive allocated intervention (n= 0)

Allocated to intervention (n= 31)

♦ Received allocated intervention (n= 31)

♦ Did not receive allocated intervention (n= 0)

Analysed (n = 26)
♦ Excluded from analysis due to insufficient biopsy material (n= 1)

♦ Gene expression results deviated strongly from the others (n = 1)

Randomized (n= 61)

**Data collection (n= 53)**

♦ Lost to follow-up due to personal circumstances (n = 1)

♦ Discontinued intervention due to diagnosis of cancer (n = 2)
